# *Eleutherococcus senticosus* Fruit Extract Stimulates the Membrane Potential of the Trachea and Small Intestine in Rabbits

**DOI:** 10.3390/molecules30204041

**Published:** 2025-10-10

**Authors:** Filip Graczyk, Elżbieta Piskorska, Dorota Gawenda-Kempczyńska, Krystian Krolik, Jakub Gębalski, Dorota Olszewska-Słonina, Aneta Kondrzycka-Dąda, Magdalena Wójciak, Orazio Taglialatela-Scafati, Robert Verpoorte, Daniel Załuski

**Affiliations:** 1 Department of Pharmaceutical Botany and Pharmacognosy, Ludwik Rydygier Collegium Medicum, Nicolaus Copernicus University, 9 Marie Curie-Skłodowska Street, 85-094 Bydgoszcz, Poland; 2 Department of Pathobiochemistry and Clinical Chemistry, Ludwik Rydygier Collegium Medicum, Nicolaus Copernicus University, 9 Marie Curie-Skłodowska Street, 85-094 Bydgoszcz, Poland; 3 University of Social Sciences, 9 Sienkiewicza Str., 90-113 Łódź, Poland; 4 Department of Analytical Chemistry, Medical University of Lublin, 4a Chodzki Str., 20-093 Lublin, Poland; 5 Department of Pharmacy, School of Medicine and Surgery, University of Naples Federico II, Via Montesano 49, 80132 Naples, Italy; 6 Natural Products Laboratory, Institute of Biology, Leiden University, 2300 RA Leiden, The Netherlands

**Keywords:** *Eleutherococcus senticosus*, trachea, small intestine, transepithelial electric potential, epithelial barrier

## Abstract

Background: *Eleutherococcus senticosus* (Rupr. et Maxim.) Maxim., widely used in Russian and Chinese traditional medicine for its anti-inflammatory activity, contains bioactive compounds capable of stabilizing epithelial function and reducing inflammation. Despite prior research on its effects in the colon, the impact and mechanism of action of *E. senticosus* fruit extract on epithelial tissues of the upper digestive and respiratory tract remains unexplored. Objectives: This study aimed to evaluate the influence of *E. senticosus* fruit extract on the transepithelial electrical potential and resistance in the tracheal and small intestinal epithelium of rabbits. In addition, the chemical composition of the extract was also profiled by the means of UHPLC-DAD-MS. Methods: Tissue segments from the trachea and small intestine of New Zealand white male rabbits were examined using the Ussing chamber technique. Three concentrations of *E. senticosus* fruit extract (0.001, 0.1, 10 mg/100 mL) were applied, and changes in transepithelial electrical potential (dPD) and resistance (R) were recorded. Chemical analysis of the extract was conducted using UHPLC-DAD-MS. Results: For the first time, we have discovered that the *E. senticosus* extract increased membrane resistance in tracheal tissue, suggesting enhanced barrier integrity. In contrast, a slight decrease in resistance was observed in small intestinal tissue. UHPLC-DAD-MS confirmed the presence of chlorogenic acid, dicaffeoylquinic acids, quercetin derivatives, and myo-inositol, compounds known for their antioxidant, anti-inflammatory, and membrane-stabilizing effects. Conclusions: The differential response of respiratory and intestinal epithelium to the *E. senticosus* extract highlights its tissue-specific action and supports its traditional use in the prevention and treatment of diseases characterized by epithelial barrier dysfunction, such as asthma, COPD, and Crohn’s disease.

## 1. Introduction

*Eleutherococcus senticosus* (Rupr. et Maxim.) Maxim., known as Siberian ginseng, is a plant used in Eastern medicine for its adaptogenic and immunostimulating properties [1]. For centuries, it has been used in traditional Chinese medicine as a means of strengthening the body, improving immunity and prolonging life. In Europe, its properties began to be intensively studied in the 1970s and 1980s, and it has now become a popular adaptogen used in disease prevention and as a means of improving the body’s performance. This plant contains numerous bioactive compounds, such as eleutherosides, flavonoids, phenolic acids and lignans, which have anti-inflammatory, immunomodulatory and antioxidant effects [2,3]. Particular attention is paid to eleutheroside B (syringin) and eleutheroside E (syringaresinol), more present in roots of the plant, which affect the permeability of cell membranes and enzymatic activity. The extracts from *E. senticosus* fruit have anti-inflammatory, antioxidant and epithelial cell ionic metabolism regulating effects [4,5].

Previous studies on the effects of *E. senticosus* fruit extract have mainly focused on the large intestine, demonstrating its ability to modify transepithelial sodium and potassium ion flux and stabilize membrane potential, especially in epithelial tissues of the distal digestive system. Importantly, its ability to inhibit hyaluronidase activity has also been demonstrated, which may be important in the treatment of inflammation and protection of the epithelium from degradation [6]. The resting membrane potential plays a key role in regulating ionic homeostasis, neural conduction, and the body’s immune response [7]. In particular, disruptions in the membrane potential in the trachea and small intestine are associated with diseases such as asthma, chronic obstructive pulmonary disease (COPD), irritable bowel syndrome, and Crohn’s disease [8]. In the context of the trachea and small intestine, research is very limited, although there is a need for the evidence to prove that adaptogens may modulate the activity of ion channels in these tissues [9].

The epithelium of the trachea and small intestine plays a key role in the homeostasis of the body, acting as a protective barrier separating the external environment from the internal structures of the body. In the respiratory system, the proper functioning of the tracheobronchial epithelium is crucial for defense against pathogens and pollutants [10,11]. Disturbances of this barrier, typical of bronchial asthma and chronic obstructive pulmonary disease (COPD), lead to increased epithelial permeability and increased inflammation [12,13]. In the digestive system, damage to the tight junctions of the intestinal epithelium and excessive inflammatory response is associated with inflammatory bowel diseases, such as Crohn’s disease [14].

In the context of the above, plants with anti-inflammatory and ion transport modulating properties, such as *E. senticosus*, may have potential application in the protection and regeneration of these epithelial structures. However, until now, there was no data on the effect of *E. senticosus* on the transepithelial electric potential in the trachea and small intestine. Since both the respiratory and gastrointestinal epithelium show significant differences in structure and ion transport mechanisms, it was necessary to extend the research in this direction.

The evaluation of transepithelial electric potential (PD), along with the measurement of transepithelial electrical resistance (R), enables the analysis of ion movement across epithelial-covered tissue. This method helps to investigate how various substances, such as the ES extract, influence the transportation of sodium, chloride, and other ions, while preserving the functional integrity of living tissue in its native responsive state [15,16].

In order to support the use of the ES fruits as a new drug material, this study is aimed to evaluate the effect of three different concentrations of *E. senticosus* fruit extract (0.001 mg/100 mL, 0.1 mg/100 mL and 10 mg/100 mL) on the membrane potential of the trachea and small intestine. It is expected to provide undiscovered, new results-based evidence on the possible use of *E. senticosus* in the therapy of respiratory and digestive system diseases, in particular diseases associated with epithelial barrier and ion transport disorders.

## 2. Results and Discussion

### 2.1. The Effect of the Extract on the Transepithelial Electric Potential in the Distal Section of the Trachea and Small Intestine

Examining the pathways involved in sodium transport sheds light on the shifts occurring at the cellular surface upon interaction with external factors. Variations in the electrical charge of the analyzed tissue sample illustrate physiological processes that take place following exposure to various compounds, including the ES extract.

The measurement of electrical resistance after each stimulation and its relatively constant value confirms the maintenance of the integrity and reactivity of the tested tissue fragments at the individual stages of each experiment. To our present understanding, this constitutes the first documentation of the effects of *Eleutherococcus senticosus* fruit extract on transepithelial ion transport in the rabbit’s small intestine and trachea epithelium. The results obtained showed that the extract significantly affects changes in the transepithelial electric potential (dPD) and electrical resistance (R) of isolated fragments of the rabbit trachea (Table 1) and small intestine (Table 2). Examining the pathways involved in sodium transport sheds light on the shifts occurring at the cellular surface upon interaction with external factors. Variations in the electrical charge of the analyzed tissue sample mirror physiological processes that take place following exposure to various compounds, including the ES extract.

Although the application of three different concentrations (0.001, 0.1 and 10 mg/100 mL) did not show any significant changes in dPD values, especially in the small intestine (all dPD values remained at 0.00 mV, with minimal IQR variations), significant changes in electrical resistance values were noted. In the case of tracheal tissue, a gradual increase in electrical resistance R was observed in response to successive applications of the extract, reaching maximum values after the application of the highest concentration of INT-10 (Me = 227.25 Ω × cm^2^; IQR = 293.75). This phenomenon may indicate a strengthening effect of extract on the integrity of the epithelial barrier in the respiratory tract, but without simultaneous modulation of the direct flow of sodium ions, which would be visible as changes in dPD.

On the other hand, an opposite trend was observed in the case of the small intestine—electrical resistance after the use of extract (especially INT-0.1 and INT-10) did not increase, and actually decreased compared to the reference values. This may indicate differences in the response of the intestinal epithelium to the active compounds of the ES extract, which should be associated with a different expression of ion transporters and the structure of the barrier compared to the respiratory tissue.

The interpretation of these results suggests that *E. senticosus* extract does not employ a classical depolarizing or hyperpolarizing effect over the measured 60 min period, but rather acts in a stabilizing or strengthening way on the epithelial barrier, which is important in the context of diseases with accompanying damage to epithelial integrity, such as asthma, COPD, IBS, or Crohn’s disease.

Our present analysis was specifically designed to examine electrogenic ion transport, offering only a partial perspective on the full scope of colonic physiology. Despite these inherent limitations, the observed alterations should be interpreted as representative of in vivo conditions. The similarity between ion transport mechanisms in the rabbit and human intestine is well-established [17], thereby supporting the validity of our findings as reliable of physiological responses in humans.

### 2.2. Ultra-High-Performance Liquid Chromatography (UHPLC)

Plants produce numerous compounds responsible for their pharmacological activity, which are unmatched by synthetic compounds. However, within the wide spectrum of phytochemicals found in a single species, only a small compartment is often responsible for the activity. One of the most dominant groups of plant-based compounds are polyphenols. Complementing the physiological data, UHPLC-DAD-MS analysis confirmed the presence of numerous compounds with anti-inflammatory and antioxidant activity, including phenolic acids, flavonoids, lignans, and their glycosides, many of which are known for their significant pharmacological properties. Although their effect on ion transport was not directly assessed in this study, the literature suggests their involvement in protection against oxidative stress and modulation of the inflammatory response, which could be a potential explanation for the observed increase in R without change in dPD.

**Figure 1:**
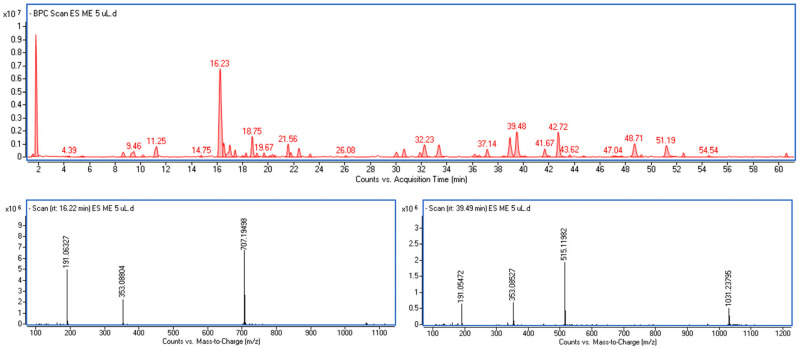
The UHPLC chromatographic profile of *Eleutherococcus senticosus* fruit extract. Identified compounds include chlorogenic acid, dicaffeoylquinic acids, eleutheroside E, and quercetin derivatives. Peaks marked correspond to retention times and compound identities listed in Table 3. The lower panel shows representative MS spectra of the predominant constituents identified as chlorogenic acid and 1,5-dicaffeoylquinic acid.

The components of the extract were identified based on MS and UV-Vis spectra extracted from individual peaks recorded on the chromatogram (Figure 1). Most of them exhibited characteristic absorption spectra with a maximum at around 325–330 nm and a pseudomolecular fragment ion at *m*/*z* [M–H]^−^ = 179, (Figure S1 in Supplementary Materials), which is typical for caffeic acid derivatives. These compounds were therefore assigned as derivatives of caffeic acid.

The most abundant among them was 5-caffeoylquinic acid commonly known as chlorogenic acid (*m*/*z* [M–H]^−^ = 353), together with several isomeric forms of dicaffeoylquinic acids (diCQA) with *m*/*z* [M–H]^−^ = 515, differing in the position of substitution of caffeoyl residues on the quinic acid core. The isomeric forms of diCQA showed identical spectral characteristics (Figure S2); however, they differed in their retention times. Three of them were identified by direct comparison with authentic standards, namely 3,5-dicaffeoylquinic acid, 1,5-dicaffeoylquinic acid, and 4,5-dicaffeoylquinic acid, while the remaining compounds were described as dicaffeoylquinic acid isomers.

Among the detected compounds, the greatest pharmacological significance is attributed to chlorogenic acid (25.22 mg/g), dicaffeoylquinic acids (including 3,5-diCQA—3.81 mg/g, 4,5-diCQA—2.38 mg/g and 1,5-diCQA—5.61 mg/g) and flavonols like quercetin. Chlorogenic acid, by far the most abundant compound in the extract, is known for its antioxidant, anti-inflammatory and metabolism-regulating properties [18,19,20]. Its high content in the extract may explain the observed epithelium-stabilizing effect and increased electrical resistance in the tracheal tissue, probably resulting from the strengthening of intercellular connections and protection against oxidative stress. Dicaffeoylquinic acids, such as 3,5-diCQA, 1,5-diCQA and 4,5-diCQA, present in amounts exceeding 10 mg/g, have potent free radical scavenging properties and inhibit the activity of inflammatory enzymes such as lipoxygenases. Their synergistic action with other phenolic components may support the protection of the epithelial barrier under conditions of inflammatory stress and help stabilize membrane potential [21,22]. It is proved that molecules such as chlorogenic acid, 5-caffeoylquinic acid, protocatechuic acid, and caffeic acid alleviated intestinal barrier damage. In addition, chlorogenic acid increased transepithelial electrical resistance and reduced paracellular permeability; furthermore, by inhibiting endoplasmic reticulum stress and increasing mucus secretion it enhances the integrity of the mucosal barrier [18].

Eleutheroside E is a lignan typical of the *Eleutherococcus* genus, known for its adaptogenic and immunomodulatory effects. The presence in the *intractum* may be important in the long-term modulation of the immune response and may explain the lack of acute changes in dPD while improving membrane resistance. However, compared to root extracts, its quantity in the extract is negligible.

Based on extracted ion method, LC-MS analysis also detected the presence of myo-inositol (4.84 ± 0.13 mg/g) (range of *m*/*z*: 179.055–179.0.057; estimated formula: C_6_H_12_O_6_) and trace amounts of mannitol (range of *m*/*z*: 181.071–181.073; estimated formula: C_6_H_14_O_6_), typical compounds from the group of sugar alcohols, known for their osmoregulatory and membrane-stabilizing effects. Myo-inositol plays an important role in cell signal transduction, involved in the regulation of ion channels, membrane function and cellular response to oxidative stress. Myo-inositol is a natural, cyclic-sugar-alcohol, which plays a fundamental role in many cellular and physiological processes. It is a precursor for the formation of key signaling compounds, such as phosphatidylinositols (PIs) and inositol triphosphate (IP3), which participate in signal transduction within cells. It also plays crucial role in the protection of the epithelial barrier by stabilizing epithelial integrity and regulating the activity of tight junction proteins. Moreover, this sugar reduces the production of reactive oxygen species (ROS), as well as inhibits the production of pro-inflammatory cytokines such as TNF-α, IL-6 and IL-1β and regulates the function of ion channels and calcium pathways, which is key to maintaining membrane potential. The content of myo-inositol in *E. senticosus* extract may suggest its participation in the observed stabilization of tracheal epithelial membrane resistance and in the absence of acute changes in dPD. Moreover, as a natural osmolyte, myo-inositol may support the maintenance of cellular homeostasis under stress conditions, which is an additional adaptogenic mechanism of this preparation [23,24,25,26]. In turn, mannitol, although present in much lower amounts, may contribute to the stabilization of epithelial integrity under stress conditions. Its presence may enhance the effect of other components of the extract in the context of cell membrane physiology; moreover, it is proved that this molecule alone, can contribute to increased drug transport across the respiratory epithelia, such as cephalosporin molecules [27], and also intracellular transport of plasmids containing genetic material [28]. As a known osmoprotective and antioxidant agent, mannitol can help maintain membrane potential by preventing damages and supporting epithelial cell resilience. Including these primary metabolites in the assessment of the biological activity of the *E. senticosus* preparation provides a broader perspective on the mechanisms of its action—not only as a source of strong antioxidants, but also as a complex stabilizer of membrane function and regulation of cell signaling [29,30,31,32].

Research conducted by Xu et al. indicates that hypersensitivity and allergic responses are linked to an upregulation of epithelial sodium channel activity and the infiltration of immunocompetent cells into electrically altered tissue regions, potentially initiating an immune reaction [33]. Damage to the respiratory epithelium and inhalation can accelerate its penetration and effects on the body. In the digestive tract, the rate of penetration is affected by the degree of food filling and the presence of inflammation [34,35].

Plants synthesize a wide range of secondary metabolites responsible for their pharmacological effects, frequently demonstrating higher biological diversity and potency compared to synthetic compounds. However, within the complex phytochemical profile of a single species, a limited number of molecules are typically responsible for the observed biological activity. Consequently, the observed effects are unlikely to be attributed to a single compound but rather to the combined or synergistic actions of multiple bioactive constituents. To the best of our knowledge, this study provides the first evidence of the effects of *E. senticosus* fruit extract on transepithelial ion transport in the rabbit small intestine and trachea epithelium, suggesting a previously uncharacterized mechanism by which *E. senticosus* fruit extract may differently influence ion transport across those epithelial tissues. This effect may be linked to the strong antioxidant potential and high total polyphenol and flavonoid content previously discussed. Given the current scarcity of studies addressing this phenomenon, our results highlight a novel and promising direction for further investigation.

The observed effect of *E. senticosus* fruit extract on transepithelial resistance, especially the stabilizing impact on tracheal tissue, highlights its potential utility in clinical settings associated with epithelial barrier dysfunction. This is particularly relevant in diseases such as chronic obstructive pulmonary disease (COPD), and asthma, where epithelial integrity is known to be disrupted, as well as in Crohn’s disease. In COPD, a condition associated with chronic airway inflammation and epithelial remodeling, reinforcement of epithelial resistance as seen in the tracheal model aligns with the need to protect the airways from oxidative stress and microbial invasion. The results here support the potential of *E. senticosus* to enhance barrier integrity in the respiratory tract, a key therapeutic target in COPD [36,37]. In Crohn’s disease, characterized by transmural intestinal inflammation and increased paracellular permeability, the weakening of intestinal resistance observed in this study may reflect a pathological mechanism similar to what occurs in active disease. Polyphenolic compounds found in the extract—especially chlorogenic acid and dicaffeoylquinic derivatives—may offer protective effects by stabilizing tight junctions and modulating inflammation, potentially aiding in intestinal barrier repair [38].

Notably, the current findings contrast with our previous research [6], where we investigated the effect of the same extract on the distal colon using a comparable experimental model. That study reported significant hyperpolarization of the colonic epithelium following treatment, suggesting enhanced electrogenic sodium transport. This response, absent in the small intestine in the present study, emphasizes regional differences in epithelial reactivity, possibly due to variations in transporter expression, microbiota composition, or oxidative environment. These comparative insights underline the tissue-specific pharmacodynamics of *E. senticosus* and reinforce the necessity of tailoring therapeutic strategies to the anatomical and physiological context of the target tissue.

## 3. Materials and Methods

### 3.1. Plant Material

The *E. senticosus* (Rupr. et Maxim.) Maxim. fruits were used to prepare the extract according to the methods presented in our previous research [6]. The authenticity of the plant material was confirmed based on the analysis of morphological features by prof. D. Załuski and HPLC-DAD test, the results of which were compared with reference substances. The fully ripe fruits were collected in the Medicinal and Cosmetic Plant Garden in Bydgoszcz, Poland, in September 2023 (53°07′36.55″ N, 18°01′51.64″ E) and deposited at the Department of Pharmaceutical Botany and Pharmacognosy of the Collegium Medicum in Bydgoszcz (Cat. Nr. ES 10/2023).

Briefly, the extract preparation process included maceration of fresh, fully ripe fruits (20 g) in 100 mL of 40% (*v*/*v*) ethanol for 30 days, at room temperature and without access to light (according to the Galen’s terminology this type of extract is called—*intractum*). After maceration, the extract was filtered using Whatman No. 4 filter paper. The solvent was then removed by evaporation under reduced pressure at 45 °C, and the sample was frozen (−20 °C) and lyophilized. The obtained lyophilizate was stored in a refrigerator at 4 °C. The metabolomic profile of the extract was determined and confirmed using UHPLC [9].

### 3.2. The Effect of the Extract on the Transepithelial Electric Potential in the Section of the Rabbit Trachea and Small Intestine

#### Experimental Procedure

The study employed the Ussing technique to assess electrophysiological parameters in isolated fragments of epithelial tissue of the small intestine and trachea, accordingly to the methods presented in our previous research [6]. These tissue samples were positioned in a modified Ussing chamber, which facilitated both mechanical and mechanical-chemical stimulation to enable synchronized measurement of multiple electrophysiological parameters while allowing tissue stimulation with the tested extract samples.

The tests were performed using epithelial tissue of the small intestine and trachea. Tissue segments were obtained from 10 male New Zealand white rabbits. The animals were approximately 15 ± 3 weeks old and weighed 2.5 to 4.0 ± 0.5 kg. They were housed individually in cages under controlled environmental settings, kept at 18 ± 2 °C and 65 ± 5% humidity. A 12-h light–dark cycle was introduced with a light intensity of 50 lux, provided with the water and certified fodder for rabbits ad libitum. The study was approved by the Local Committee for Ethical Experiments on Animals of the Universities of Bydgoszcz (approval number 23/2009).

Anesthesia was induced using 4% isoflurane distributed in the carbon dioxide. In each case, death was confirmed. The organs were removed and the intestine was immediately cleaned and rinsed with Ringer’s solution. Whole tracheae were used for the study. The examined fragments were tested immediately after collection; the samples were then longitudinally opened and cut into fragments of approximately 2.0 cm^2^. These specimens were incubated in designated solutions before being placed horizontally between the two halves of the Ussing chamber, which were filled with the corresponding incubation fluid. To prevent tissue damage, every sample was placed on a paper filter.

To stimulate the tissue sample, the inner surface of the tissue wall was gently rinsed with experimental solutions using a needle attached to a peristaltic pump. The nozzle was placed 2 mm from the surface, delivering the rinse at a pressure of approximately 6 Pa. Every stimulus lasted 15 s, with the temperature of 37 °C carried out for the whole experiment.

The Ussing chamber system, which allows for precise recording of electrical changes in the epithelium of the trachea and small intestine were used. The two halves of the Ussing chamber were connected via two pairs of agar passages to Ag/AgCl electrodes, which were then linked to an EVC 4000 amplifier (World Precision Instruments, Sarasota, FL, USA). Ag/AgCl electrodes were used for electrochemical analysis of the membrane potential, during the experiment, the difference in transepithelial potential (dPD [mV]) and tissue resistance (Ω×cm^2^) were recorded to assess the integrity and electrokinetic properties of the epithelium. Data acquisition, processing, and analysis were performed using the MP150 computer system (Biopac System, Goleta, CA, USA) in conjunction with AcqKnowledge 3.8.1 software.

### 3.3. Chemicals and Solutions

All the chemical substances employed were of analytical reagent grade, and distilled water was used. To determine the effect of *E. senticosus* on the electrical properties, the following solutions were prepared, of which three different concentrations of ES fruit extract were used:

- 0.001 mg/100 mL (INT-0.001);
- 0.1 mg/100 mL (INT-0.1);
- 10 mg/100 mL (INT-10);
- The Ringer solution (RH): 147.2 mM Na^+^, 4.0 mM K^+^, 2.2 mM Ca^2+^, 2.6 mM Mg^2+^, 155.6 mM Cl^−^, pH 7.4 (POCH, Poland).

The selected concentrations of ES fruit extract (0.001, 0.1, and 10 mg/100 mL) were chosen to explore a broad range of doses in order to evaluate their potential effects on the studied tissues. This approach allowed us to capture both very low and relatively high concentrations, which provides insight into the possible therapeutic window. Three concentrations of the extract were added to solutions on one side of the epithelium and measurements were then taken for 60 min, recording changes in transepithelial potential and membrane resistance for each concentration. A series of at least five replicates was performed in each study group.

### 3.4. Ultra-High-Performance Liquid Chromatography (UHPLC-DAD-MS)

The extract was examined using an ultra-high-performance liquid chromatograph (UHPLC) Infinity Series II equipped with a DAD detector and an Agilent 6224 ESI/TOF mass spectrometer (Agilent Technologies, Santa Clara, CA, USA). The separation was performed on a Kinetex C18 column (Phenomenex, Torrance, CA, USA) with a length of 150 mm, an internal diameter of 2.1 mm, and a particle size of 1.7 µm. The column temperature was maintained at 30 °C, and the mobile phase flow rate was set at 0.2 mL/min. The injection volume was 5 µL.

The mobile phase consisted of water with 0.05% formic acid (solvent A) and acetonitrile with 0.05% formic acid (solvent B). Gradient elution was performed according to the following program: 0–8 min from 98% A to 93% A, 8–15 min from 93% A to 88% A, 15–29 min from 88% A to 85% A, 29–40 min from 85% A to 80% A, 40–80 min from 80% A to 55% A. LC–MS conditions: The drying gas temperature was set at 325 °C, with a gas flow rate of 8 L/min. The nebulizer pressure was maintained at 30 psi, while the capillary voltage was set to 3500 V. The skimmer voltage was 65 V, and the fragmentor voltage was applied at 200 V and 280 V. Ions were acquired in the range of 100 to 1200 *m*/*z* in negative ions. Identification was based on UV-VIS spectra and MS data. The spectral data and retention times were compared with commercially available standards (Sigma-Aldrich, St. Louis, MO, USA), or components were tentatively identified based on formulas generated by MassHunter software (version 10.0). An acceptable mass error was defined as not exceeding 5 ppm. Quantification was performed using calibration curves prepared from standard solutions in methanol. Each sample was injected three times. Details of the quantitative analysis, including detection parameters, concentration ranges, and calibration equations, are provided in the Supplementary Materials (Table S1).

### 3.5. Statistical Analysis

The collected data was statistically analyzed using the Statistica 12 program, with the Wilcoxon test for repeated measures, which allowed for the assessment of the significance of differences between the control samples and the samples treated with the extract. The statistical significance level was set at *p* < 0.05. The obtained results were expressed in units proper for a specified parameter (for R—Ω*cm^2^, for dPD—mV) and presented as median (Me) and quartile range (IQR) values.

## 4. Conclusions

To sum up, our current study demonstrates that *Eleutherococcus senticosus* fruit extract exhibits a differential effect on epithelial tissue, enhancing resistance in respiratory epithelium while slightly reducing it in intestinal model. The ES extract at higher concentrations (10 mg/100 mL) enhanced tracheal resistance, while showing a reduction in intestinal resistance. These findings support its potential utility in conditions marked by epithelial barrier dysfunction. This effect may be attributed to the content of the phytochemicals like chlorogenic acid, dicaffeoylquinic acid and myo-inositol, which could help alleviate oxidative and inflammatory stress.

From that perspective, the data indicate that *E. senticosus* extract could serve as a supportive therapy in diseases such as Crohn’s disease, COPD, and asthma, where restoration or maintenance of barrier integrity is critical. Further studies are needed to validate these effects in vivo and to explore dose-dependency and chronic applications, including the major phytochemical compounds. It may also focus on evaluating isolated active compounds or defined fractions to verify their individual roles in the observed effects.

## Figures and Tables

**Table 1:** The effect of *Eleutherococcus senticosus* extract on changes in transepithelial electrical potential (dPD) and resistance (R) in isolated rabbit tracheal tissue. Values presented as median (Me) and interquartile range (IQR). RS—controls, INT—different concentrations of extract (mg/100 mL).

*n* = 20	dPD [mV] *n* = 20		R [Ω×cm^2^] *n* = 20	
	Median	IQR	Median	IQR
RS-1	−0.64 a	1.97	216.75	140.00
INT-0.001	0.00 ab	0.72	224.25	177.50
RS-2	−0.24 bc	1.39	225.75	206.00
INT-0.1	0.00 b	0.42	228.00	244.25
RS-3	−0.33 b	0.53	227.25	276.00
INT-10	0.00 c	0.41	227.25	293.75
RS-4	−0.31 b	0.28	227.25	302.75

a,b,c—statistically significant differences marked with different letters (*p* < 0.05).

**Table 2:** The effect of *Eleutherococcus senticosus* extract on changes in transepithelial electric potential (dPD) and resistance (R) in isolated rabbit small intestine tissue. Values presented as median (Me) and interquartile range (IQR). RS—controls, INT—different concentrations of extract (mg/100 mL).

*n* = 20	dPD [mV] *n* = 20		R [Ω×cm^2^] *n* = 20	
	Median	IQR	Median	IQR
RS-1	0.00 ab	0.06	66.25	41.00
INT-0.001	0.00 ab	0.06	78.75	54.75
RS-2	0.00 a	0.06	66.50	38.75
INT-0.1	0.00 ab	0.00	58.75	35.00
RS-3	0.00 b	0.12	67.75	38.00
INT-10	0.00 ab	0.20	58.00	31.25
RS-4	0.00 ab	0.12	61.00	37.25

a,b,c—statistically significant differences marked with different letters (*p* < 0.05).

**Table 3:** The phytochemical profile of *Eleutherococcus senticosus* fruit extract identified by UHPLC-DAD-MS.

Rt (min)	Observed Ion Mass [M–H]^−^/(Characteristic Fragments)	Error (ppm)	Formula	Identified	Concentration (mg/g)
1.66	179.05588	−1.29	C_6_H_12_O_6_	myo-inositol	4.84 ± 0.13
1.68	181.07122	−2.98	C_6_H_14_O_6_	mannitol	+
1.79	191.05656	2.33	C_7_H_12_O_6_	quininc acid	11.81 ± 1.01
6.67	299.07761 (137)	1.23	C_13_H_16_O_8_	hydroxybenzoic acid hexoside	+
6.80	315.07254 (153)	1.22	C_13_H_16_O_9_	protocatechuic acid hexoside	+
8.70	153.01932 (109)	−0.08	C_7_H_6_O_4_	protocatechuic acid *	0.41 ± 0.02
11.25	353.08842 (191, 179)	1.73	C_16_H_18_O_9_	neochlorogenic acid *	1.61 ± 0.08
14.27	387.12931 [M+HCOOH]^−^/(179)	−0.93	C_16_H_22_O_8_	coniferin	+
14.82	451.12473 (289)	0.32	C_21_H_24_O_11_	catechin hexoside	0.23 ± 0.02
15.59	289.07182	0.20	C_15_H_14_O_6_	catechin *	0.25 ± 0.02
16.18	353.08804 (191, 179)	0.66	C_16_H_18_O_9_	chlorogenic acid *	25.22 ± 1.12
16.88	579.1333 (285)	−3.87	C_26_H_28_O_15_	cyanidin derivative	2.75 ± 0.12
16.99	179.03532	1.88	C_9_H_8_O_4_	caffeic acid *	0.19 ± 0.01
18.75	353.08847 (191, 179)	1.88	C_16_H_18_O_9_	cryptochlorogenic acid *	0.61 ± 0.04
19.12	415.16043	−1.3	C_19_H_28_O_10_	unknown	+
19.7, 20.4, 21.6, 24.5 **	335.07756 (179)	0.95	C_16_H_16_O_8_	caffeoylshikimic acid isomer	0.96 ± 0.06
21.6,22.5, 24.1, 24.9 **	367.10366 (191, 173)	0.55	C_17_H_20_O9	feruloylquinic acid isomer	0.43 ± 0.03
26.06	787.26391 [*m*/*z*+HCOOH]^−^	−3.65	C_34_H_46_O_18_	eleutheroside E *	0.20 ± 0.02
30.66	609.14703	1.51	C_27_H_30_O_16_	quercetin 7-*O*-rutinoside *	0.47 ± 0.03
31.91	609.14667 (463, 300)	0.92	C_27_H_30_O_16_	quercetin 3-*O*-rutinoside *	0.25 ± 0.02
32.26	463.08857 (300)	0.80	C_21_H_20_O_12_	quercetin 3-*O*-galactoside *	0.41 ± 0.03
33.38	463.08876 (300)	1.21	C_21_H_20_O_12_	quercetin 3-*O*-glucoside *	0.43 ± 0.03
36.16	515.11967 (353, 191, 179)	0.33	C_25_H_24_O_12_	dicaffeoylquinic acid isomer	0.45 ± 0.02
37.14	515.11902 (353, 191, 179)	−0.93	C_25_H_24_O_12_	dicaffeoylquinic acid isomer	0.62 ± 0.03
38.76	515.11916 (353, 191, 179)	−0.66	C_25_H_24_O_12_	3,5-dicaffeoylquinic acid *	3.81 ± 0.18
39.48	515.11981 (353, 191, 179)	0.60	C_25_H_24_O_12_	1,5-dicaffeoylquinic acid *	5.61 ± 0.27
42.72	515.12037 (353, 191, 179)	1.69	C_25_H_24_O_12_	4,5-dicaffeoylquinic acid *	2.38 ± 0.12
48.36	515.12099 (353, 191, 179)	2.89	C_25_H_24_O_12_	dicaffeoylquinic acid isomer	0.15 ± 0.01
48.71	207.06715 (179, 161, 133)	4.17	C_11_H_12_O_4_	caffeic acid derivative	0.39 ± 0.02
51.19	301.03656	3.92	C_15_H_10_O_7_	Quercetin *	0.17 ± 0.01
60.58	577.13572 (193, 385)	0.99	C_30_H_26_O_12_	diferulic acid derivative	+

Rt—retention times; +—detected but not quantified because lack of appropriate standard. *—compounds confirmed with reference standards; **—different retention times indicate that some isomers were present in the samples, and the quantification results represent the sum of all isomers.

## Data Availability

The original contributions presented in this study are included in the article/Supplementary Materials. Further inquiries can be directed to the corresponding author.

## Supplementary Materials

The following supporting information can be downloaded at: https://www.mdpi.com/article/10.3390/molecules30204041/s1, Figure S1. BPC chromatogram (red line), extracted ion chromatogram at *m*/*z* range of 179.03–179.04 (blue line), and DAD chroma-23; Figure S2. MS spectra and DAD spectra extracted from peaks identified as chlorogenic acid and different isomers of 27; dicaffeoylquinic acid found in Eleutherococcus senticosus fruit extract chromatogram at λ = 320 nm (green line) of Eleutherococcus senticosus fruit extract; Table S1. Calibration data for quantitative analysis.

## Abbreviations

The following abbreviations are used in this manuscript:

dPD	changes in the transepithelial electrical potential difference after 15 s mechanical and mechanical-chemical stimulation (mV)
ES	*Eleutherococcus senticosus*
INT	*Eleutherococcus senticosus* extract
Me	median
n	number of specimens isolated from the rabbit trachea and small intestine wall
*p*	statistically significant difference at *p* < 0.05
PD	transepithelial electrical potential difference (mV)
R	transepithelial electrical resistance (Ω/cm^2^)
RH	Ringer solution
RS	control stimulation
