# Supplementary material for: Eleutherococcus senticosus Fruit Extract Stimulates the Membrane Potential of the Trachea and Small Intestine in Rabbits
Source: Molecules. 2025 Oct 10;30(20):4041. doi: 10.3390/molecules30204041 (PMC12566354; doi:10.3390/molecules30204041)
Supplement: Supplementary file 1 [file molecules-30-04041-s001.zip › molecules-3873729-supplementary.pdf]

molecules

Supplementary materials

Eleutherococcus senticosus fruit extract stimulates the membrane potential of the trachea and small intestine in rabbits – from traditional use to explanation of the mechanism of action

Filip Graczyk[1]*, Elżbieta Piskorska[2], Dorota Gawenda-Kempczyńska[1], Krystian Krolik[1], Jakub Gębalski[1], Dorota Olszewska-Słonina[2], Aneta Kondrzycka-Dąda[3], Magdalena Wójciak[4], Orazio Taglialatela-Scafati[5], Rob Verpoorte[6], Daniel Załuski[1]

[1]Department of Pharmaceutical Botany and Pharmacognosy, Ludwik Rydygier Collegium Medicum, Nicolaus Copernicus University, 9 Marie Curie-Skłodowska Street, 85-094 Bydgoszcz, Poland
[2]Department of Pathobiochemistry and Clinical Chemistry, Ludwik Rydygier Collegium Medicum, Nicolaus Copernicus University, 9 Marie Curie-Skłodowska Street, 85-094 Bydgoszcz, Poland
[3]University of Social Sciences, 9 Sienkiewicza Str., 90-113 Łódź, Poland
[4]Department of Analytical Chemistry, Medical University of Lublin, 4a Chodzki Str., 20-093 Lublin, Poland
[5]Department of Pharmacy, School of Medicine and Surgery, University of Naples Federico II, Via Montesano 49, 80132 Naples, Italy
[6]Natural Products Laboratory, Institute of Biology, Leiden University, 2300 RA Leiden, The Netherlands

*Corresponding Author: Filip Graczyk, phone: (+48) 795672587, E-mail: filip.graczk@gmail.com

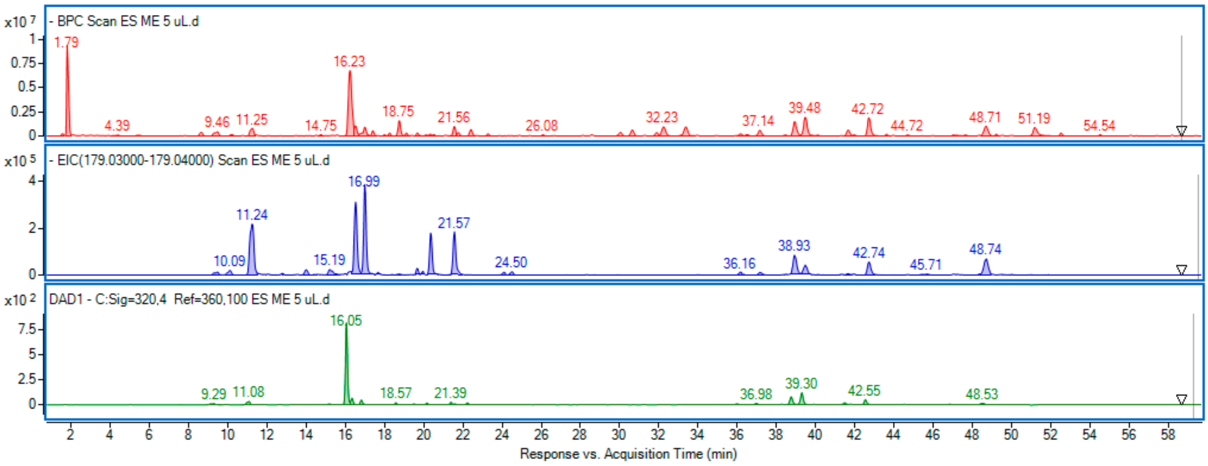

Figure S1. BPC chromatogram (red line), extracted ion chromatogram at m/z range of 179.03-179.04 (blue line), and DAD chromatogram at λ=320 nm (green line) of *Eleutherococcus senticosus* fruit extract.

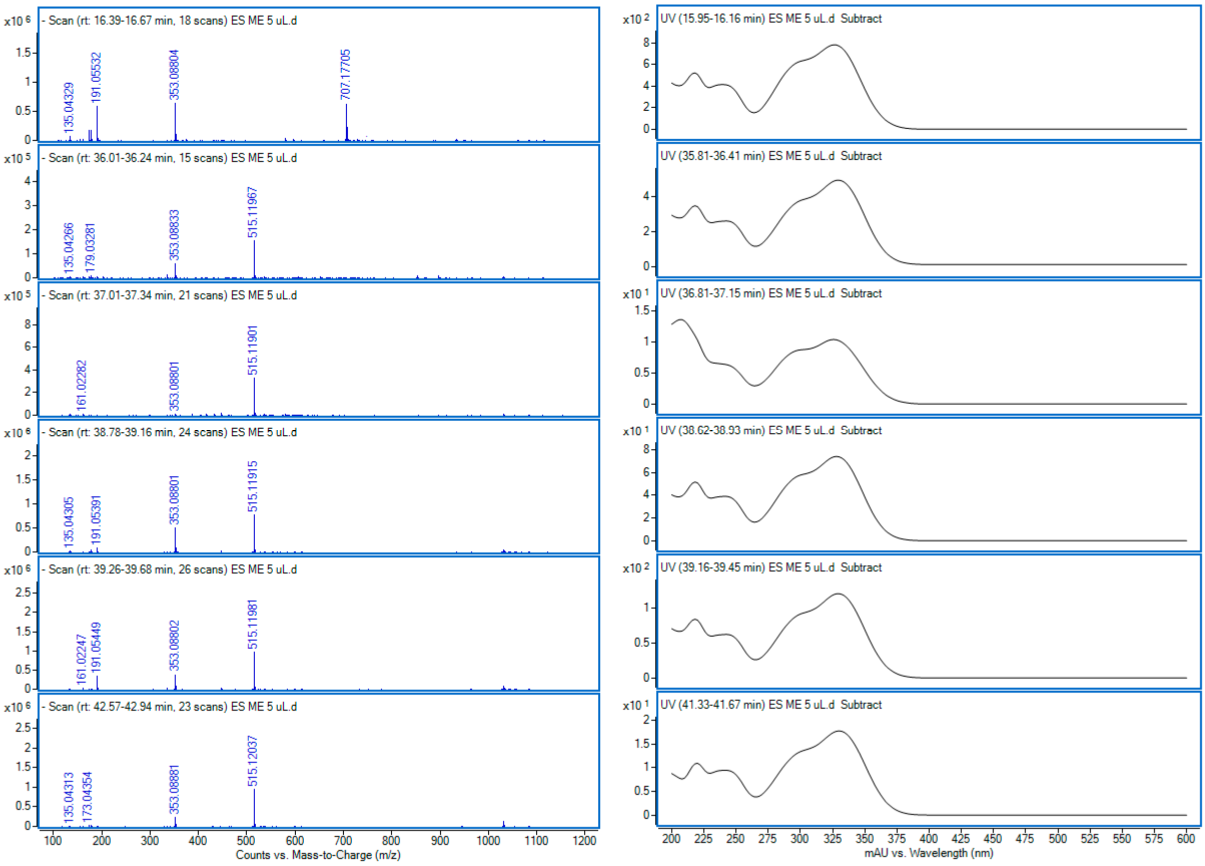

Figure S2. MS spectra and DAD spectra extracted from peaks identified as chlorogenic acid and different isomers of dicaffeoylquinic acid found in *Eleutherococcus senticosus* fruit extract.

Table S1. Calibration data for quantitative analysis.

Compound	Detection	Range (mg/mL)	Equation	Compound calculated
Protocatechuic acid	DAD 254 nm	0.0005–0.005	86531x – 1.7	protocatechuic acid
Caffeic acid	DAD 320 nm	0.0002–0.002	148648x – 20.9	caffeic, caffeoylshikimic acid
Catechin	DAD 280 nm	0.0005–0.005	6351.7x - 0,2	catechin and catechin hexoside
Neochlorogenic acid	DAD 320 nm	0.0012–0.012	44066x – 1.3	neo- and cryptochlorogenic acid
Chlorogenic acid	DAD 320 nm	0.015–0.150	44376x – 5.3	chlorogenic acid
Ferulic acid	DAD 320 nm	0.0001–0.001	77913x – 2.4	feruloylquinic acids
Cyanidin	DAD 520 nm	0.002–0.020	6363x – 1.2	cyanidin derivative
Eleutheroside E	DAD 230 nm	0.0002–0.002	345.6x – 0.3	eleutheroside E
Quercetin 3-O-rutinoside	DAD 350 nm	0.0005–0.005	29737x – 1.9	quercetin 3- and 7- O-rutinoside
Quercetin 3-O-glucoside	DAD 350 nm	0.0003–0.003	82919x – 7.7	quercetin 3-O-glucoside
Quercetin 3-O-galactoside	DAD 350 nm	0.0003–0.003	85214x – 8.5	quercetin 3-O-galactoside
Quercetin	DAD 350 nm	0.0001–0.001	59905x – 1.6	quercetin
3,5-dicaffeoylquinic acid	DAD 320 nm	0.003–0.030	48391x – 87.1	3,5- and 1,5-dicaffeoylquinic acid
3,5-dicaffeoylquinic acid	DAD 320 nm	0.0003-0.003	41087x – 20.0	isomers of dicaffeoylquinic acid
4,5-dicaffeoylquinic acid	DAD 320 nm	0.0025–0.025	51109x – 12.3	4,5-dicaffeoylquinic acid
myo-inositol	MS range: 179.05-179.06	0.015–0.035	1244915448x + 16358490	myo-inositol
quininc acid	MS range: 191.05-191.06	0.020-0.080	1138252699x + 18535879	quininc acid

Disclaimer/Publisher's Note: The statements, opinions and data contained in all publications are solely those of the individual author(s) and contributor(s) and not of MDPI and/or the editor(s). MDPI and/or the editor(s) disclaim responsibility for any injury to people or property resulting from any ideas, methods, instructions or products referred to in the content.
